# Nonhost diversity and density reduce the strength of parasitoid–host interactions

**DOI:** 10.1002/ece3.2191

**Published:** 2016-05-18

**Authors:** Rachel Kehoe, Enric Frago, Catherin Barten, Flurin Jecker, Frank van Veen, Dirk Sanders

**Affiliations:** ^1^ Centre for Ecology & ConservationUniversity of ExeterPenrynCornwallTR 10 9EZUK; ^2^ Laboratory of EntomologyWageningen UniversityP.O. Box 8031WageningenNL‐6700 EHThe Netherlands; ^3^ CIRADUMR PVBMTF‐97410Saint‐PierreLa RéunionFrance; ^4^ Community EcologyInstitute of Ecology and EvolutionUniversity of BernBaltzerstrasse 6Bern3012Switzerland

**Keywords:** Aphids, associational effects, indirect interactions, searching behavior, stability, trait‐mediated

## Abstract

The presence of nonprey or nonhosts is known to reduce the strength of consumer– resource interactions by increasing the consumer's effort needed to find its resource. These interference effects can have a stabilizing effect on consumer–resource dynamics, but have also been invoked to explain parasitoid extinctions. To understand how nonhosts affect parasitoids, we manipulated the density and diversity of nonhost aphids using experimental host–parasitoid communities and tested how this affects parasitation efficiency of two aphid parasitoid species. To further study the behavioral response of parasitoids to nonhosts, we tested for changes in parasitoid time allocation in relation to their host‐finding strategies. The proportion of successful attacks (attack rate) in both parasitoid species was reduced by the presence of nonhosts. The parasitoid *Aphidius megourae* was strongly affected by increasing nonhost diversity with the attack rate dropping from 0.39 without nonhosts to 0.05 with high diversity of nonhosts, while *Lysiphlebus fabarum* responded less strongly, but in a more pronounced way to an increase in nonhost density. Our experiments further showed that increasing nonhost diversity caused host searching and attacking activity levels to fall in *A. megourae*, but not in *L. fabarum*, and that *A. megourae* changed its behavior after a period of time in the presence of nonhosts by increasing its time spent resting. This study shows that nonhost density and diversity in the environment are crucial determinants for the strength of consumer–resource interactions. Their impact upon a consumer's efficiency strongly depends on its host/prey finding strategy as demonstrated by the different responses for the two parasitoid species. We discuss that these trait‐mediated indirect interactions between host and nonhost species are important for community stability, acting either stabilizing or destabilizing depending on the level of nonhost density or diversity present.

## Introduction

Interactions between species define the network structure of communities and are key components that drive ecological and evolutionary processes (Fontaine et al. 2011). Species often interact directly, for example, through predation or parasitism, but ecologists are increasingly aware of the importance of indirect interactions whereby at least a third species serves as a mediator (Wootton 1994). Although indirect interactions are considered to be of equal importance to direct interactions in explaining community structure and functions, they are often hard to observe, and their impact on ecological processes remain difficult to predict (Janssen et al. 1998). Indirect effects can be mediated by changes in the density of an intermediate species or by changes in their traits (physical, chemical or behavioral) (Wootton 1993; Menge 1995). An example of a well‐known trait‐mediated indirect interaction is the impact of predators on plant biomass through their effect on herbivore feeding behavior (Beckerman et al. 1997). This interaction is indirect because the interaction between the predator and the plant is mediated through the herbivore, and is trait‐mediated because the effect is mediated through fear‐induced changes in feeding behavior in herbivore prey, which causes the prey to spend less time foraging. In particular in terrestrial ecosystems, trait‐mediated indirect effects have been shown to influence population dynamics, species coexistence, top‐down trophic cascades, food web structure, and soil mineralization (see Gastreich 1999; Peacor and Werner 2001; Schmitz et al. 2004; Schmitz 2008; van Veen and Godfray 2012 for a review).

Within insect herbivore communities, apparent competition and trophic cascades as examples of indirect effects have been well studied (Morris et al. 2004; van Veen et al. 2006). Despite this, we lack experimental knowledge about the relevance of indirect effects that arise when natural enemies face complex communities (Tylianakis and Romo 2010). This is a common situation for most consumers, and we know, for example, that insect herbivores can benefit from being embedded in diverse plant communities, where the efficiency of their natural enemies is reduced (Moreira et al. 2016). This phenomenon is known as associational resistance, as the proximity of a resource to another species can deceases the resource's vulnerability to predation and can thus increase its fitness (Barbosa et al. 2009). Although relatively less understood, associational resistance can also apply to the presence of nonprey or nonhost species in the community. In both situations, species (plant or insect herbivore) diversity increases the physical and chemical complexity of the environment where predators forage. This ultimately benefits prey because discriminating hosts or prey in such complex scenarios can have a marked influence on consumers' rate of discovering hosts or prey, and therefore upon their consumption rate (Wootton 1992, 1993; Vos et al. 2001; Grabowski 2004; Kratina et al. 2007; van Veen and Godfray 2012).

Currently, there is great interest in understanding the forces that allow for stability and prevent extinctions in complex communities (Vos et al. 2001; van Veen et al. 2005; Sanders and van Veen 2012). Theoretical modeling suggests that increasing parasitoid–host diversity produces a more stable community (Vos et al. 2001), and experimental studies have demonstrated that indirect interactions are key to understand such long‐term stability (Sanders et al. 2013, 2015). In communities with multiple herbivore species, specialist natural enemies in particular, can stabilize community dynamics by preventing any herbivore from dominating the community. However, for this equilibrium to be maintained, interactions with nonhost aphids are required as they reduce parasitoid efficiency and can thus prevent parasitoids overexploiting their hosts (van Veen et al. 2005). In an experiment where both the diversity and density of nonprey rotifers was manipulated, Kratina et al. (2007) showed that these changes altered the functional response of a predatory flatworm. Although the same principle may operate in terrestrial systems, there is little evidence available. In order to understand the behavioral mechanism behind these nonhost effects, we need to use a model system that allows for the following of an individual's changes in behavior.

Aphids and their parasitoids are an ideal model system to study the effects of nonhost presence, due to their easily tractable trophic interactions (with successfully parasitized aphids becoming mummies) and short generation times (Hassell 2000). Additional to this, the behavioral mechanisms behind community interactions can be easily observed (Frago and Godfray 2013). As with predators, parasitoids have intricate behavioral strategies to locate their host, displaying searching behavior, host handling time, and species‐specific attack rates. The oviposition success of a parasitoid is influenced by many parameters including the quality of the habitat that the host lives in, its spatial structure, host encounter rates, and the way in which the parasitoid handles the host (Doutt 1959). Waage (1979) developed a behavioral model for the parasitoid *Nemeritis canescens* to describe the optimal time a parasitoid might spend in an area, related, amongst other things, to number of hosts, and oviposition success. This model describes how an individual getting poor returns in an environment should leave the patch to forage in another. Understanding the behavioral repertoire that different parasitoid species use to locate their hosts will help us understand how these species will be affected when foraging in complicated scenarios. We use the two parasitoid species *Lysiphlebus fabarum* and *Aphidius megourae* as they respond differently to similar situations in multigenerational community experiments. In these experiments, the highly specialist parasitoid *A. megourae* appears to be more sensitive to changes in relative host abundance than *L. fabarum* (Sanders et al. 2013). In using these two different parasitoid species, we expect a difference in results due to their differing levels of host specificity and searching strategies. This aphid–parasitoid model system has previously been used in multigenerational community level experiments, where the extinction of carnivores was observed while their prey species remained present, suggesting the carnivore extinctions were caused by an increase in nonhosts in the environment (Sanders et al. 2015). The present study provides a specific set of experiments a mechanistic understanding of such nonhost effects as shown to be of high importance for community stability in previous studies.

In this study, we use three separate experiments to test whether different levels of nonhost density and diversity affect (1) the attack rate of parasitoids, and to further understand the behavioral changes behind these effects (2) the proportion of time spent by parasitoids foraging and (3) the change in parasitoid behavior with increasing time in the environment. We hypothesize (1) that increasing nonhost density and diversity will reduce the attack rate of two parasitoids as they spend more time and energy in finding their hosts among the nonhosts with a stronger response in the more specialized species *A. megourae*, (2) a change in foraging behavior of parasitoids by either a reduction or an increase in parasitoid activity dependant on the presence of nonhost and (3) a change in parasitoid behavior according to the time spent in complex environments, with searching effort either dropping off quickly in high diversity nonhost situations due to lower returns for energetic output, invoking a “giving‐up” strategy, or maintaining at the same level if host patches are normally hard to find, and so individuals are less willing to leave.

## Material and Methods

### Study system

Our system is comprised of plant–aphid–parasitoid communities consisting of *Vicia faba* L. (var. the Sutton), three aphid species *Megoura viciae* (Buckton 1876), *Myzus persicae* (Sulzer), and *Aphis fabae* (Scopoli), and two parasitoids, *A. megourae* (Stary, 1965) and *L. fabarum* (Marshall, 1896), parasitizing *M. viciae* and *A. fabae*, respectively. *A. fabae* and *M. persicae* were used as nonhost species for *A. megourae*, whereas for *L. fabarum, M. viciae* and *M. persicae* were used. Prior to the experiments, aphids and parasitoids were kept on *V. faba* in a controlled temperature room at 20 ± 1 °C with a 16:8‐h light:dark cycle. All aphids used in the experiments were 3 days old, this being a parasitoid's preferred host age (Stary 1988).

### Attack rate experiment

To test whether increasing nonhost density and diversity reduces attack rate (hypothesis 1), pots with three 2‐week‐old bean plants were set up with the following aphid compositions aimed at manipulating nonhost density and diversity in a full factorial design, replicated nine times for each of the two parasitoid species (see Table 1): The following levels of density and diversity were tested in this experiment with seven different nonhost treatments: (1) host only 20 nonhosts, (2 + 4) Low Density, Low Diversity 20 hosts 40 nonHosts (for two different nonhost species A or B), (3 + 4) High Density Low Diversity 20 hosts 80 nonhosts (A or B), (6) High Diversity Low Density 20 hosts 20 nonhost A 20 nonhost B, (7) High Diversity High Density 20 hosts 40 nonhost A 40 nonhost B. In all treatments, aphid densities were kept far below the carrying capacity, therefore avoiding potentially confounding effects through different levels of competition between aphids.

**Table 1:** Experimental design showing different treatments with relative densities of hosts and absolute diversity of nonhosts in the three experiments: Attack Rate, Foraging Activity, and Change of Behaviour Over Time. Hosts for the parasitoids *A. megourae* and *L. fabarum* were the aphids *M. viciae* (*Mv*) and *A. fabae* (*Af*), respectively. Nonhosts for *A. megourae* were *A. fabae* (*Af*) and *M. persicae* (*Mp*)*,* and for *L. fabarum* were *M. viciae* (*Mv*) and *M. persicae* (*Mp*)

Treatment	Relative host abundance	Parasitoid	Aphid numbers in the different experiments					
			Attack Rate		Foraging Activity		Change of Behavior	
			Host	NonHost	Host	Nonhost	Host	Nonhost
1. Host Only	100	*A. megourae*	20 *Mv*	–	*6 Mv*		*6 Mv*	
		*L. fabarum*	20 *Af*	–	*6 Af*		*6 Af*	
2. Low Density Low DiversityNonHost A	33	*A. megourae*	20 *Mv*	40 *Af*	*6 Mv*	12 *Af*	*6 Mv*	*12 Af*
		*L. fabarum*	20 *Af*	40 *Mv*	*6 Af*	12 *Mv*	*6 Af*	*12 Mv*
3. High Density Low DiversityNonHost A	25	*A. megourae*	20 *Mv*	80 *Af*	*6 Mv*	*24 Af*		
		*L. fabarum*	20 *Af*	80 *Mv*	*6 Af*	24 *Mv*		
4. Low Density Low DiversityNonHost B	33	*A. megourae*	20 *Mv*	40 *Mp*	*6 Mv*	12 *Mp*		
		*L. fabarum*	20 *Af*	40 *Mp*	*6 Af*	12 *Mp*		
5. High Density Low DiversityNonHost B	25	*A. megourae*	20 *Mv*	80 *Mp*	*6 Mv*	24 *Mp*		
		*L. fabarum*	20 *Af*	80 *Mp*	*6 Af*	24 *Mp*		
6. Low Density High DiversityNonHost A and B	33	*A. megourae*	20 *Mv*	20 *Af* and 20 *Mp*	*6 Mv*	*6 Af* and *6 Mp*	*6 Mv*	*6 Af* and *6 Mp*
		*L. fabarum*	20 *Af*	20 *Mv* and 20 *Mp*	*6 Af*	*6 Mv* and 6 *Mp*	*6 Af*	*6 Mv* and *6 Mp*
7. High Density High DiversityNonHost A and B	25	*A. megourae*	20 *Mv*	40 *Af* and 40 *Mp*	*6 Mv*	*12 Af* and 12 *Mp*		
		*L. fabarum*	20 *Af*	40 *Mv* and 40 *Mp*	*6 Af*	12 *Mv* and 12 *Mp*		

Each pot was placed in a gauze population cage (48 cm^3^) and left for 2 h to allow aphids to settle. A mated female parasitoid was then released into each cage and left for 48 h. Cages were kept in a controlled temperature room arranged in spatial blocks, each containing one replicate of each treatment. After removal of the parasitoids, cages were kept in the controlled temperature room for 11 days until aphids had reached maturity, or developed into mummies (which allows the precise calculation of the proportion of successful attacks).

### Foraging activity experiment

To explore hypothesis 2, whether nonhost treatments affect the parasitoids' efficiency in detecting and attacking hosts through changes in foraging behavior, a second experiment that allowed the observation of parasitoid behavior was performed. Single bean leaves were placed in vented petri dishes with the same relative aphid compositions as in the previous experiment, but with fewer aphids (See Table 1 for nonhost treatments): (1) Host Only 6 hosts, (2 + 4) Low Density Low Diversity 6 hosts 12 nonhosts (nonhost species A or B), (3 + 5) Low Density High Diversity 6 hosts 24 nonhosts (A or B), (6) High Diversity Low Density 6 hosts 6 nonhost A 6 nonhost B, (7) High Density High Diversity 6 hosts 12 nonhost A 12 nonhost B. Each treatment was replicated 15 times for each parasitoid species. After the aphids had settled on the leaf, one female parasitoid was released in each dish and observed for 10 min. During this time, we noted the amount of time each parasitoid was active. Parasitoids were considered as active when they were walking on the leaf or attempting to oviposit, and considered inactive when they were resting or preening.

### Change of behaviour over time experiment

To test hypothesis 3 and get a deeper understanding of the specific behavior of *A. megourae* and *L. fabarum* after having spent time in the presence of nonhosts*,* we performed a third experiment. A single bean leaf was placed in a petri dish. The leaves were infested with aphids to obtain the following three nonhost treatments (1) Host Only 6 hosts (2) Low Density Low Diversity 6 hosts 12 nonhosts (6) Low Density High Diversity 6 hosts 6 nonhost A 6 nonhost B. After the aphids had settled on the leaf, one female of either *A. megourae* or *L. fabarum* was released into the corresponding petri dish. Every 30 sec over a period 10 min we recorded whether the parasitoid was resting or active (see above for definition of behavior) to detect whether they change behavior over time. Each treatment was replicated 20 times for each parasitoid species.

### Statistical analyses

We used linear mixed effects models with binomial error structure to test for the impact of nonhost density and diversity on the proportion of successful host attacks for the two different parasitoid species *L. fabarum* and *A. megourae*. As response variable, we included the number of mummified aphids relative to those that were not parasitized. Nonhost density (0, 2 × host density, 4 × host density) as well as nonhost diversity (0,1,2 species) were analyzed as continuous variables. These, along with parasitoid identity (*L. fabarum* or *A. megourae*) to test whether species responded differently to the treatments were included as fixed effects and block was included as a random effect.

The behavioral “Foraging Activity” experiment was analyzed in a similar way, with the proportion of parasitoid active time relative to their inactive time combined as response variable tested in models with a binomial error structure. In these models, the same fixed and random factors as above were included.

In the “Change of Behaviour Over Time” experiment, we focussed on the impact of nonhost diversity on parasitoid behavior. The resting versus active behavior of single parasitoids was observed over 10 min at 20 time steps (every 30 sec) and included as response variable. To account for the repeated measurement (nonindependence) of each parasitoid individual we included the term “individual nested in block” as a random factor. Treatment (nonhost diversity 0,1,2), time and time squared were included as fixed factors. Time squared was included as covariate to account for nonlinearity over time. We used lme4 package (Bates et al. 2013) in R version 3.2.2 (R Core Team 2015).

## Results

### Attack rate

**Figure 1:**
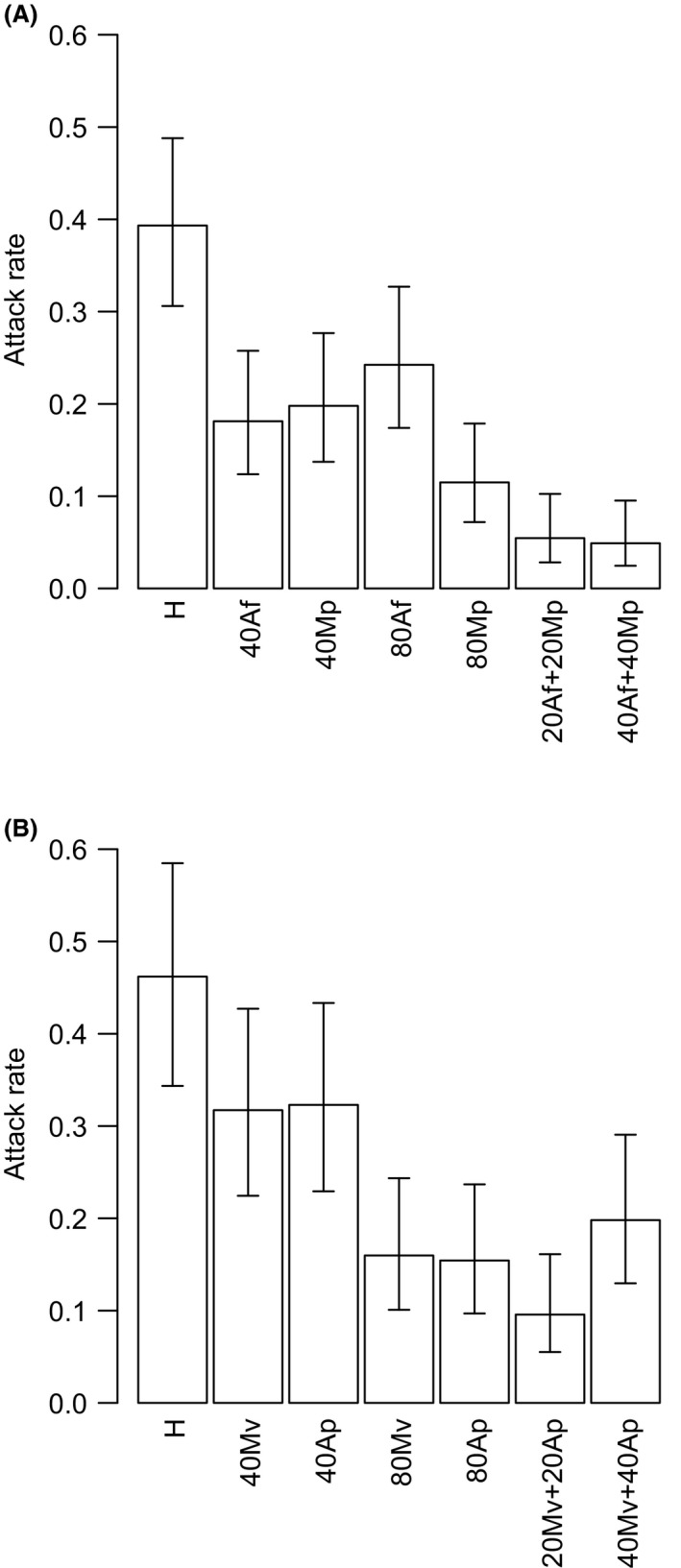
Attack rate, defined as proportion of successful attacks (with 95% confidence intervals) by the parasitoids *Aphidius megourae* (A) and *Lysiphlebus fabarum* (B) under control conditions with 20 hosts only (H) and in treatments with different density and diversity of nonhost species (see Table 1). Hosts for *A. megourae* and *L. fabarum* were the aphids *M. viciae* (*Mv*) and *A. fabae* (*Af*), respectively. Nonhosts for *A. megourae* were *A. fabae* (*Af*) and *M. persicae* (*Mp*)*,* and for *L. fabarum*:* M. viciae* (*Mv*) and *M. persicae* (*Mp*). Treatments were replicated nine times for each parasitoid, for statistical analysis see Table 2.

The attack rate of both parasitoid species, *L. fabarum* and *A. megourae*, was negatively influenced by the presence of nonhosts. The parasitoid species responded differently to nonhost density and diversity, demonstrated by a significant interaction between both variables *nonhost density* and *nonhost diversity* with *parasitoid species* (Fig. 1, Table 2). The parasitoid *A. megourae* responded more strongly to an increase in nonhost diversity than in nonhost density, with the opposite pattern being displayed by the parasitoid *L. fabarum*. The attack rate in *A. megourae* dropped from 0.39 in the Host Only treatment, to 0.1 in the High Density Low Diversity nonhost treatment (*Mp*) and to a markedly low attack rate of 0.05 in both the Low Density High Diversity and High Density High Diversity nonhost treatments (20*Af *+ 20*Mp* and 40*Af *+ 40*Mp*) (Fig. 1A, Table 2). *L. fabarum* responded to an increase of nonhost density by reducing its attack rate from 0.46 in the Host Only to 0.15 in the High Density nonhost treatments as well as, but to a lesser degree, in the High Density High Diversity nonhost treatment (40*Mp *+ 40*Mv*) reducing its attack rate to 0.20 (Fig. 1B, Table 2).

**Table 2:** Results of mixed effects model with binomial error structure for the proportion of successful attacks by the two parasitoid species on their respective hosts. Nonhost density (0, 2 × host density, 4 × host density) as well as nonhost diversity (0,1,2) were both manipulated at three levels, parasitoid species with two levels (with *A. megourae* as intercept), while block was included as random factor. Treatments were replicated nine times for each parasitoid species

	Estimate	Std. error	*z* Value	*P*
Intercept	−0.37	0.16	−2.34	0.019
Nonhost density	0.04	0.14	0.32	0.747
Nonhost diversity	−1.24	0.18	−6.75	<0.001
Parasitoid species	0.40	0.21	1.06	0.291
Nh‐density × parasitoid species	−0.39	0.18	−2.14	0.032
Nh‐diversity × parasitoid species	0.75	0.23	3.31	<0.001

### Foraging activity experiment

**Figure 2:**
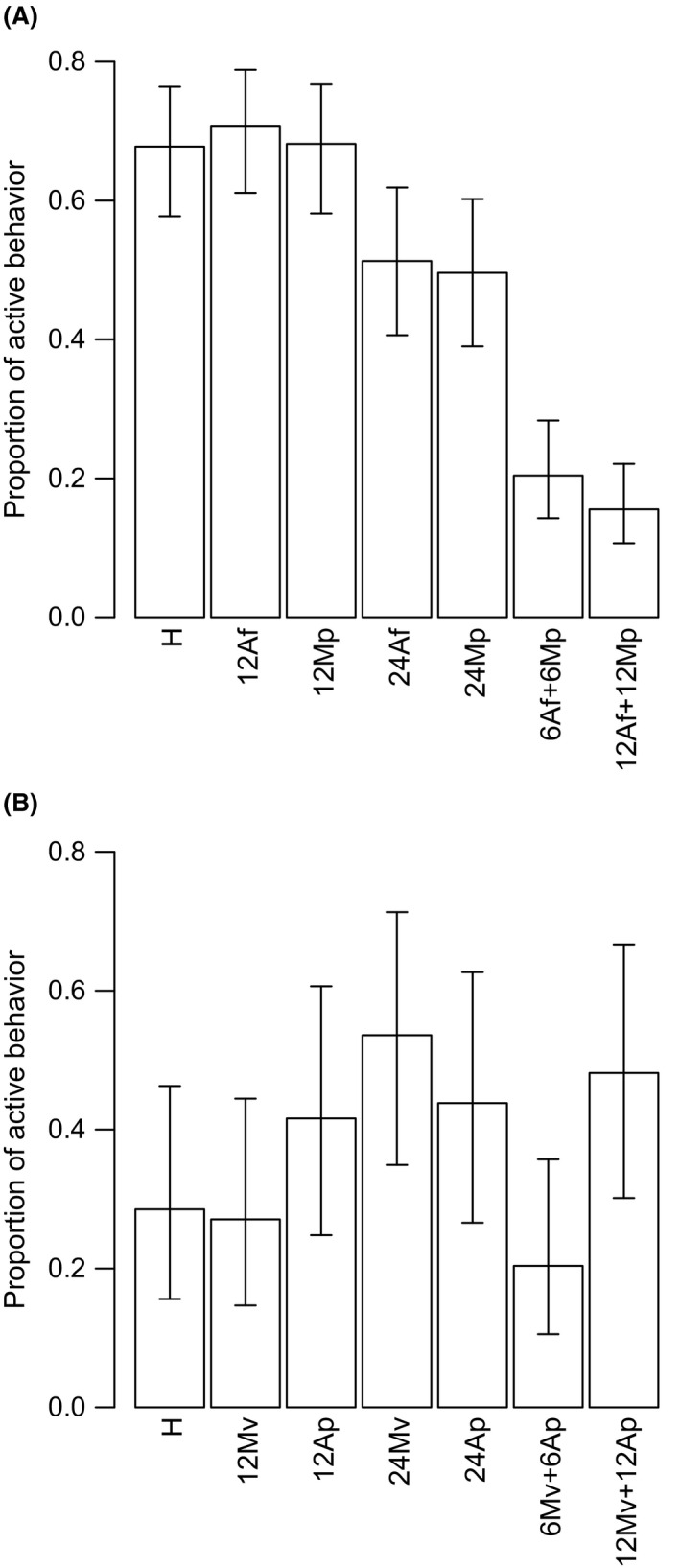
Proportion of time spend searching for hosts and attacking (with 95% confidence intervals) for the parasitoids *Aphidius megourae* (A) and *Lysiphlebus fabarum* (B) during 10 min with six hosts only (H) and in treatments with different density and diversity of nonhost species (see Table 1). Hosts for *A. megourae* and *L. fabarum* were *M. viciae* (*Mv*) and *A. fabae* (*Af*), respectively. The nonhosts used for *A. megourae* were *A. fabae* (*Af*) and *M. persicae* (*Mp*)*,* and for *L. fabarum* were *M. viciae* (*Mv*) and *M. persicae* (*Mp*). Means and confidence intervals are based on 15 replicates for each treatment, for statistical analysis see Table 3.

Both density and diversity of nonhosts had an impact on the proportion of active behavior shown by *A. megourae,* but not by *L. fabarum* (Fig. 2, Table 3). The proportion of active behavior in *A. megourae* dropped from 0.75 in the Host Only treatment to 0.50 in the High Density Low Diversity nonhost treatment. Increasing nonhost diversity markedly decreased parasitoid activity rate to 0.20 in the Low Density High Diversity nonhost treatment and to 0.18 in the High Density High Diversity nonhost treatment (Fig. 2A). The behavior of *L. fabarum* was not influenced by the presence of nonhosts; however, it displayed a consistently lower proportion of active behavior than *A. megourae* (Fig. 2, Table 3). The interaction between nonhost density, nonhost diversity and parasitoid species was significant, showing that at high levels of nonhost density and diversity, the impact of nonhost presence on parasitoid activity was dependent of the parasitoid species, with *A. megourae* dropping to a rate of 0.18 while *L. fabarum* was unaffected (displaying a trend in the other direction).

**Table 3:** Results of mixed effects model with binomial error structure for the proportion of active behavior displayed by the two parasitoid species in the different nonhost treatments (Fig. 2). Nonhost density (0, 2 × host density, 4 × host density) as well as nonhost diversity (0,1,2) were both manipulated at three levels, species with two levels (with *A. megourae* as intercept), while block was included as random factor. Each treatment was replicated 15 times per parasitoid species

	Estimate	Std. error	*z* Value	*P*
Intercept	0.9680	0.3139	3.08	0.002
Nonhost density	0.7093	0.3016	2.35	0.019
Nonhost diversity	−0.4429	0.3484	−1.27	0.203
Parasitoid species	−1.8174	0.4271	−4.26	<0.001
Nh‐density × Nh‐diversity	−0.8554	0.2699	−3.17	0.001
Nh‐density × parasitoid species	−0.2087	0.4268	−0.49	0.625
Nh‐diversity × parasitoid species	−0.2737	0.4944	−0.55	0.580
Nh‐density × Nh‐diversity × parasitoid species	1.1423	0.3820	2.99	0.003

### Change of behaviour experiment

**Figure 3:**
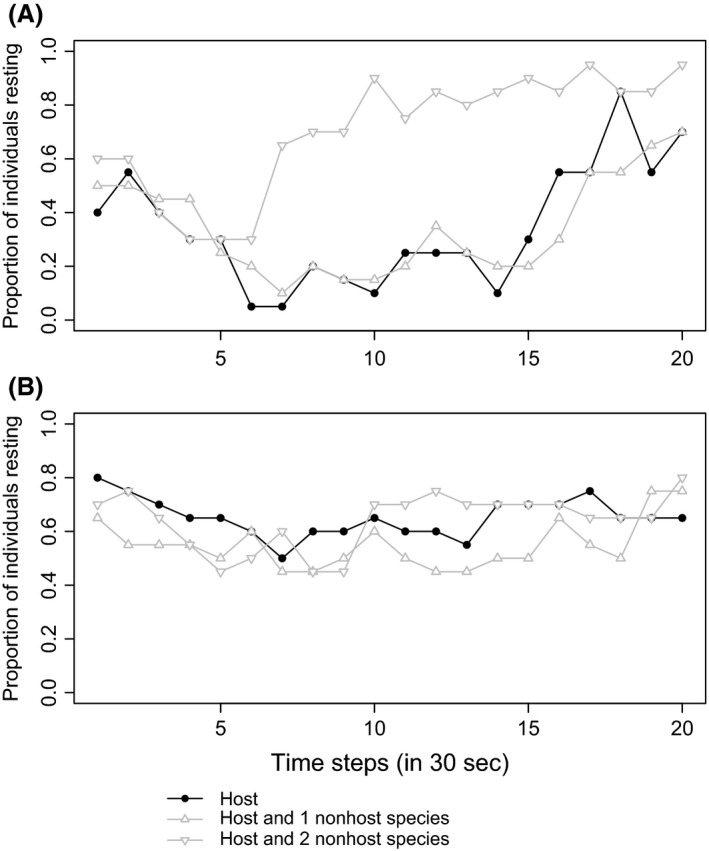
Proportion of parasitoid individuals showing resting behavior over a period of 10 min in different treatments with either hosts only, 1 nonhost species or 2 nonhost species present (see Table 1) for the parasitoids *Aphidius megourae* (A) and *Lysiphlebus fabarum* (B). One time step corresponds to 30 sec, and each treatment was replicated 20 times per parasitoid species. For statistical analysis see text.

*Aphidius megourae* and *L. fabarum* showed differing responses to the different levels of nonhost diversity in their behavioral response over time (Fig. 3). *A. megourae* showed a similar resting rate in all three treatments at the beginning of the experiment, at 0.5 ± 0.05, and all treatments gradually reduced resting rate until 2.5 min. At this point, the behavior in the Host Only treatment and the Low Density Low Diversity nonhost treatment stayed at a low resting rate. A high proportion of parasitoids (80%) in the Low Density High Diversity nonhost treatment switched to resting behavior after 5 min until the end of the experiment (treatment × time interaction, *z* = 4.557, *P* < 0.001). *L. fabarum,* however, remained at a consistent activity rate, throughout the duration of the experiment (no treatment effect: χ = 4.921, *P* = 0.0853).

## Discussion

Our experiments show (1) that attack rates of both parasitoids were reduced in response to nonhosts in the environment, with the parasitoid *A. megourae* being more sensitive to an increase in nonhost diversity, whereas the parasitoid *L. fabarum* displayed sensitivity to increasing nonhost density. We further found that (2) increasing nonhost diversity caused host searching and attacking activity levels to fall in the parasitoid *A. megourae*, but not in *L. fabarum*, and (3) that *A. megourae* changed its behavior after 3 min in the presence of higher diversity of nonhosts by increasing its resting rate, while *L. fabarum* remained at a constant resting rate. Therefore, nonhost presence and especially nonhost diversity was an important factor reducing the attack rate of parasitoids, indicating a positive trait‐mediated indirect effect of nonhosts over host survival. These interactions can be considered as an associational resistance effect, as one species indirectly benefits from the presence of another. Our study therefore confirms the results reported for a freshwater system composed of nematodes feeding on rotifers (Kratina et al. 2007) for a terrestrial plant‐based system. It further extends our understanding of these effects by including the behavioral response of parasitoids, which differs for the two species used in our study.

The remarkable difference in the response of the two parasitoid species to nonhosts is an important result and reflects the outcome of a multigenerational host–parasitoid community experiment in which the parasitoid species showed different levels of sensitivity to disturbance, with *A. megourae* being far more sensitive (Sanders et al. 2013). This could be due to their contrasting host‐finding strategies, stemming from differences in host specificity: *A. megourae* is highly specialized (monophagous) on the host *M. viciae* while *L. fabarum* is more generalistic and has a host range that includes at least 144 aphid species from 36 genera (Carver 1984). The wide host range in *L. fabarum* is likely to make this species more adapted to foraging in complex scenarios, as it can switch hosts, something that *A. megourae* is not capable of. To understand why the impact on attack rates differed between the two species, we explored the differences in behavior the parasitoids displayed in response to nonhost presence. When nonhost diversity was high, *A. megourae* demonstrated a typical “giving‐up” time strategy (Barnard 1980), where predators or parasitoids leave the resource area when host quantity is low. This is an optimal strategy in a variable environment (Marschall et al. 1989), when there is an energetic cost to foraging (Brown 1988) and therefore finding a new and better patch is the optimal strategy. *L. fabarum*, in contrast, remained at a relatively constant activity level across all nonhost treatments suggesting that this species has a different measure for patch quality and as such is more resistant to leave an environment where there are few hosts.

Nonhost presence may impact upon the effectiveness of parastioids by adding their chemical signatures to the environment. The aphid *M. vicae* releases three terpene hydrocarbons, *α*‐pinene, *β*‐pinene, and limonene, whereas both *A. fabae* and *M. persicae* release (E)‐ *β*‐farnesene, a common, highly concentrated “foundation” terpene (Francis et al. 2005) released also by the plant *V. faba* (Du et al. 1996). Hosts, with their specific hydrocarbons, may therefore become much harder to detect chemically for the parasitoids when they are surrounded by nonhosts with either additional or similar hydrocarbons. Extensive work has demonstrated that parasitoids use chemical cues from insect‐infested plants (i.e., herbivore induced plant volatiles) to locate their hosts (Vet and Dicke 1992). Relatively less is known about the chemical information parasitoids use once on the plant, and how chemical complexity affects their decision making. Evidence suggests that this complexity can have long‐term community consequences. Frago and Godfray (2014) found that the presence of chemical cues from an aphid predator in a plant affected parasitoid efficiency with consequences for the population dynamics of the aphids in those and nearby plants. This study shows that host localization for parasitoids is compromised in chemically complex environments. Such complexity can also be caused by nonhosts confusing parasitoids and indirectly protecting nearby aphids. Our study revealed the importance of nonhost diversity in altering parasitoid foraging, but future work is needed to unveil the complex chemical interactions involved in parasitoid foraging.

Host–parasitoid population dynamics often display an oscillating pattern, with larger host numbers giving rise to larger parasitoid numbers, which will in turn cause host numbers to decline, (van Veen and Godfray 2012). In this situation, if the parasitoid is very efficient this can eventually lead to local species extinctions, unless there is a refuge for the host species or a mechanism to reduce predator efficiency at high densities (Hassell 2000). One such refuge can be provided by nonhosts, giving rise to a stable system (van Veen et al. 2005). However nonhosts have also the potential to decrease stability. Using a similar community as the one we worked here, Sanders et al. (2013) studied long‐term population dynamics and cascading extinctions. This study showed that harvesting one of the parasitoid species (simulating a functional extinction where the species is still present, but at very low numbers) can cause extinctions of indirectly linked parasitoids in a community with three specialist parasitoids and three aphid hosts. These extinctions were caused via changes in the relative density of the different aphids (i.e., competition at the host level), and they occurred despite the host still being present. The hypothesis for these extinctions was that even if hosts were present, the parasitoids struggled to locate and attack their hosts due to large numbers of nonhosts. More specifically, these effects were stronger in the parasitoid *A. megourae,* which was more susceptible to the disturbance, with more extinctions than *L. fabarum*. This pattern matches the findings of the present study, and thus species‐specific response of consumers to nonhosts or nonprey can be critical for the stability of the whole community. In particular, increased nonhost diversity is an important factor that can lead to local extinctions.

Community stability requires a balance between species, and in predator–prey or parasitoid–host systems this importantly involves host‐finding strategies. Specialists such as *A. megourae* are unlikely to find a host in a patch where a high numbers of nonhosts are present, and they may thus easily leave a patch when encounters occur. The opposite might happen with generalists like *L. fabarum*, which are more likely to find a host if they carry on searching. Although in our study only two species were studied, differences in their strategies may explain the differences that we previously found at the community level. In view of the global changes imposed by human activities, the topology of many trophic webs will be altered due to environmental changes and the arrival of new species. Both scenarios can lead to an increase in nonhosts or nonprey abundance, leading to a new context for consumer–prey interactions, which could result in consumer extinctions. A behavioral understanding of these interactions is crucial to predict extinctions, especially because previous studies revealed that extinctions may happen long before the resource species goes extinct (Sanders et al. 2015).

In conclusion, nonhost effects are highly important for consumer–resource interactions, with density and especially diversity of nonhosts or nonprey being important factors. Different species are affected in different ways and to different degrees, with parasitoid strategy and host detectability both having an impact on these interactions. This study both matches and advances on previous work on nonhosts, and provides the general mechanism that shows how nonhosts can stabilize community dynamics (Vos et al. 2001) or when strong enough, even lead to extinction cascades (Sanders et al. 2013, 2015).

## Conflict of Interest

None declared.
